# Protective Effects of Iridoid Glycoside from Corni Fructus on Type 2 Diabetes with Nonalcoholic Fatty Liver in Mice

**DOI:** 10.1155/2021/3642463

**Published:** 2021-01-20

**Authors:** Dou Niu, Xue Chen, Ting Wang, Fuxing Wang, Qiusheng Zhang, Xiaochang Xue, Jiefang Kang

**Affiliations:** Key Laboratory of the Ministry of Education for Medicinal Resources and Natural Pharmaceutical Chemistry, National Engineering Laboratory for Resource Development of Endangered Crude Drugs in Northwest of China, College of Life Sciences, Shaanxi Normal University, Xi'an 710119, China

## Abstract

Type 2 diabetes mellitus (T2DM) is a common chronic metabolic disease. Accumulating evidence has demonstrated that nonalcoholic fatty liver disease (NAFLD) shares common typical features with T2DM, and they affect each other extensively. Thus, NAFLD has emerged as a novel target for T2DM prevention and care. Although Corni Fructus (CF) and its extracts have a therapeutic effect on T2DM, its effects and mechanisms on T2DM with NAFLD are far from elucidated. In this study, a mouse model of T2DM with NAFLD complication was established in ICR mice by feeding a high-fat, high-sugar (HFHS) diet and intraperitoneally injecting with a low dose of streptozotocin (STZ). Then, the effects of iridoid glycosides (IG) extracted from CF on this mouse model were investigated. We found that 4-week IG administration remarkably alleviated hyperglycemia and insulin resistance and significantly reduced inflammation, oxidative stress, and fat accumulation in the liver of T2DM with NAFLD mice. Further studies showed that IG inhibited the NF-*κ*B but enhanced the PI3K-AKT signaling pathway. In summary, these results indicated that the IG from CF has potential therapeutic effects on T2DM with NAFLD.

## 1. Introduction

Type 2 diabetes mellitus (T2DM) is a chronic metabolic disease, and the increasing global T2DM mortality rate makes it a vital topic of medical research [1]. Most of the prevailing T2DM are caused by unhealthy lifestyles, which ultimately lead to abnormal glucose and lipid metabolism caused by insulin deficiency and subsequent insulin resistance [2]. As one of the most common liver disorders worldwide, nonalcoholic fatty liver disease (NAFLD) has been indicated to be closely related to T2DM [3]. Mounting evidence supports a direct causal relationship between NAFLD and insulin resistance, and one of the most compelling evidences is that insulin resistance can be induced in several modified NAFLD mice including SREBP-1c transgenic mice [4], ob/ob, and db/db mice [5]. In addition, although the prevalence of T2DM is 20% in the general population [6], the incidence is 65%–87% in NAFLD [7]. Additionally, while NAFLD is present in 20%–30% of the general population [3], it reaches an impressive prevalence of 50%–75% of patients suffering from T2DM [8]. Not surprisingly, it has been reported that improving NAFLD could reduce the incidence of T2DM, and antidiabetic drugs could potentially treat both of these disease states simultaneously [9, 10]. Therefore, taking into account NAFLD may accelerate progress in developing effective T2DM therapy strategies.

As of 2017, there were 7.29 million people with diabetes in China, ranking second in the world [1]. Nowadays, increasing herbs and traditional drugs are being used in the treatment of T2DM [11], and among them, Corni Fructus (CF) is one of the most used. In China, CF often appears in cooking and winemaking, and it has also been listed as a famous health functional food in China. In addition, some components like morroniside, loganin, and morroniside extracted from CF have been reported to effectively lower blood glucose levels and alleviate insulin resistance in T2DM mice [12]. Earlier studies revealed that the alcohol extract of CF and its active ingredients including morroniside, loganin, and ursolic acid possess hypoglycemic effects with the ability to apparently alleviate kidney damage caused by diabetes [13]. CF may also reduce the impact of oxidative stress [14]. However, the effect of iridoid glycoside (IG) extracted from CF that alleviate the T2DM with NAFLD has not been illustrated previously.

The Phosphatidylinositide 3-kinases-AKT (PI3K-AKT) signaling pathway is a conventional signaling pathway that plays important roles in glucose uptake [15]. For example, glucose transporter type 4 (GLUT4) is an insulin-sensitive transporter which distributed mainly in muscle and adipose tissue. In the case of glucose uptake, binding of agonistic ligands like insulin triggers autophosphorylation of the tyrosine residues on the insulin receptor, which then activates the insulin receptor substrate (IRS-1). The activated IRS-1 binds to PI3K and promotes its activation. Then, PI3K accelerated Phosphatidylinositol 3,4,5-trisphosphate (PIP3) generation and AKT activation [16]. Finally, AKT triggers the translocation of GLUT4 to the plasma membrane and diffusion of glucose into the cell [17]. Thus, enhancing the PI3K-AKT signaling pathway or GLUT4 translocation represents a promising strategy for counteracting insulin resistance in diabetes and keeping glucose level homeostasis. Zhu and colleagues reported that apelin apparently improved glucose uptake in insulin-resistant 3T3-L1 adipocytes in a PI3K/Akt pathway-dependent manner and can be a potential insulin-sensitizing agent. Metformin and rosiglitazone, two antidiabetic drugs, have been reported to potently decrease blood glucose levels in the clinical treatment of diabetes via activating the AMPK pathway and enhancing GLUT4 translocation [18]. It is well known that the nuclear factor-kappa B (NF-*κ*B) signaling pathway participates in the development of hepatic steatosis, insulin resistance, fibrosis, and inflammation during NAFLD [19], but the effects are complex. For example, deletion of NF-*κ*B essential modifier (NEMO, also known as IKBKG) in liver parenchymal cells in mice impaired liver regeneration and spontaneously developed progressive NAFLD (that is, steatosis, NASH, and cirrhosis) [20]. Additionally, accumulating evidence demonstrated that crosstalk between NF-*κ*B and other signal transduction pathways including PI3K-AKT plays pivotal roles in NAFLD development [21]. Despite our previous data showing that the IG of CF has the potential to decrease blood glucose through the PI3K-AKT signaling pathway [22], whether IG can protect mice from NAFLD is still far from elucidated.

In this current study, we hypothesized that IG of CF can treat T2DM combined with NAFLD by improving T2DM through activating the PI3K-AKT pathway on one hand and ameliorating NAFLD via inhibiting the NF-*κ*B pathway on the other. To confirm this, ICR mice were fed with HFHS diet for 4 weeks, fasted for 12 h, and intraperitoneally injected with STZ (40 mg/kg) once every three days, five times in total to induce T2DM with NAFLD model. Then, the mice were treated with IG for 4 weeks. Finally, the protective effects of IG on T2DM with NAFLD and the detailed mechanism were investigated.

## 2. Materials and Methods

### 2.1. Ethics Statements

The animal studies were performed in strict accordance with the Rules for the Administration of Animal Experiments for Medical Research Purposes issued by the Ministry of Health of China and were approved by the Animal Experiment Administration Committee of The Shaanxi Normal University. Mice were raised in a dedicated SPF facility at the Laboratory Animal Center of the Shaanxi Normal University, and all efforts were made to keep pain and suffering to a minimum.

### 2.2. Reagents

**Figure 1:**
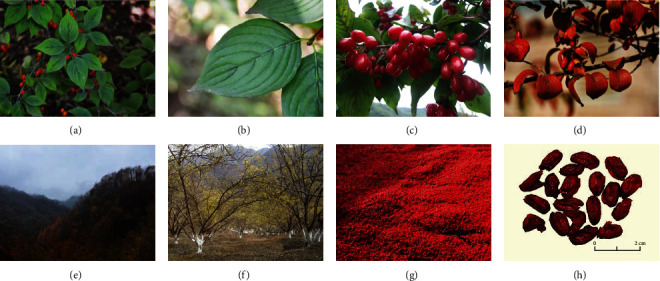
Appearance and growing environment of Corni Fructus used in this study: (a) Complete plant appearance. (b) Leaf. (c) Ripe fruits. (d) Mature leaves. (e) Growing environment. (f) Branches. (g) Picked fresh fruit. (h) Dry fruit.

CF was picked in Foping County, Shaanxi Province in October 2018. The appearance and growing environment of CF used in this study is shown in Figure 1. A blood glucose meter was purchased from Houmei De Biotechnology Co., Ltd. (Taiwan, China). The mouse total cholesterol (TC), triglyceride (TG), high-density lipoprotein cholesterol (HDL-C), low-density lipoprotein cholesterol (LDL-C), alanine aminotransferase (ALT), aspartate aminotransferase (AST), BCA, free fatty acids (FFA), malondialdehyde (MDA), total superoxide dismutase (T-SOD), total antioxidant capacity (T-AOC), and glutathione peroxidase (GSH-PX) kits were purchased from Nanjing Jiancheng Bioengineering Institute (Nanjing, China). The enzyme-linked immunosorbent assay (ELISA) kits for mouse C-reactive protein (CRP), interleukin 6 (IL-6), tumor necrosis factor-alpha (TNF-*α*), and insulin were from Shanghai Elisa Biotechnology Co., Ltd. (Shanghai, China). The antibodies against phospho-GSK3*β* (ab131097), GSK3*β* (ab131356), p65 (ab16502), PEPCK (ab181170), iNOS (1b178945), COX-2 (ab179800), phospho-TAK1 (ab192443), TAK1 (ab111096), phospho-AKT (ab38449), AKT (ab8805), phospho-IKK*β* (ab59195), phospho-JNK (ab124956), and JNK (ab179461) were obtained from Abcam (Cambridge, UK). The secondary antibodies for Western blotting analysis were from ZSGB-BIO (Beijing, China).

### 2.3. Corni Fructus Extract Preparation

The IG was extracted from CF as described previously [23]. In brief, the pitted CF was ground to fine powder after drying at 35°C. Then, 1 g of the powder was mixed with 21 ml of 80% methanol solution in a 50 ml Erlenmeyer placed in XO-200 Microwave Ultrasonic Combined Reaction System. After extraction with 641 W microwave power and 360 W ultrasonic power for 9 min, followed by three further repetitions, the extracts were combined and concentrated by vacuum distillation. Then, the extracts were resuspended in distilled water to a concentration of 0.1 g/ml and loaded into a D101 macroporous resin column preequilibrated with 50% (*v*/*v*) ethanol at a flow rate of 2 BV (bed volume)/h. IG in CF was eluted with equilibration buffer at a flow rate of 1.5 ml/min. Finally, the eluted extracts were combined and vacuum freeze-dried, and the IG products were stored at -20°C for later use.

### 2.4. Animal Treatment

Seven-week-old SPF male ICR mice weighing 18 ± 3 g were purchased from the Animal Center of Xi'an Jiaotong University (Xi'an, China) and were housed in a temperature- and humidity-controlled room with free access to standard chow and water under a 12/12 h light/dark cycle. After adaptive feeding for 7 days, the mice were randomly divided into blank group (NC) and model group (DM), in which the NC mice were given a standard chow, and the DM mice were fed with a high-fat, high-sugar (HFHS) diet (66.98% standard chow, 3% cholesterol, 10% lard, 20% sucrose and 0.02% pig bile salt) (Slac Laboratory Animal, Shanghai, China). Four weeks later, the mice were fasted for 12 h. Then, DM mice were intraperitoneally injected with STZ (40 mg/kg) once every three days, five times in total. An equal volume of sodium citrate buffer (0.1 mol/L, pH 4.2-4.5) was given to the NC mice. One week after the last injection, the mice were fasted for another 12 h, and tail vein blood was collected for fasting blood glucose (FBG) detection. The mice with FBG levels of 7.8 mmol/L or higher were considered diabetic and were used in later experiments.

The T2DM with NAFLD mice were randomly divided into five groups (*n* = 10 for each): [1] normal diet supplemented with 0.9% saline (DM group), [2] normal diet supplemented with 100 mg/kg metformin hydrochloride (PC group), (3– [5)] normal diet containing 200, 300, and 400 mg/kg of IG, designated as the IG-L, IG-M, and IG-H group, respectively. As the control, the NC group was given a normal diet. Body weights of the mice were monitored and recorded every week. After 4 weeks, the mice were fasted for 12 h, and fasting blood was collected from ophthalmic veins under anesthesia by sodium pentobarbital. Then, the mice were perfused with ice-cold saline, and the pancreas and kidneys were dissected out and fixed in 4% paraformaldehyde solution. Finally, 0.5 g of liver tissue was homogenized in 5 ml ice-cold normal saline, and the rest tissue was also fixed in paraformaldehyde solution. The serum and tissues were stored at -80°C for further analysis.

### 2.5. Measurements of Blood Glucose and Insulin-Related Indices

Fasting blood glucose (FBG) was measured using a blood glucose meter. An oral glucose tolerance test (OGTT) was performed after 12 h fasting. Briefly, the mice were orally administered with glucose solution (2 g/kg), and blood glucose levels were detected immediately prior (0 min), 30, 60, 120, and 180 min after administration. The area under the blood sugar concentration-time curve from time zero to the last measured time was calculated by the formula: AUC = (FBG + BG_30min_) × 1/4 + (BG_30min_ + BG_60min_) × 1/4 + (BG_60min_ + BG_120min_) × 1/2. Serum insulin was measured at 0 min by ELISA kit (KeYingMei Technology Co. Ltd., Beijing, China) according to the manufacturer's instructions, and the insulin sensitivity index (ISI), insulin resistance index (HOMA-IR), and insulin secretion index (HOME-*β*) were calculated from these data.

### 2.6. Measurement of Blood Lipid-Related Indices

Serum total cholesterol (TC), triglycerides (TG), high-density lipoprotein cholesterol (HDL-C), and low-density lipoprotein cholesterol (LDL-C) were measured with specific commercial kits (Beihua Kangtai Clinical Reagent Co., Ltd., Beijing, China) according to the manufacturer's recommendations. Serum free fatty acids (FFA) were measured by ELISA kits (R&D Systems, USA).

### 2.7. Liver Function Test

To detect the protective effects of IG on the liver, mouse serum was collected to determine the ALT and AST activities, which were used as biochemical indicators of hepatic injury and were measured by ALT and AST kits (Jiancheng Biotech Co., Nanjing, China) according to the manufacturer's instructions.

### 2.8. Histology Observation

Tissues were fixed in 4% neutral paraformaldehyde for 48 h and dehydrated with gradient ethanol solution, followed by embedding in paraffin. The embedded tissues were then sectioned at a thickness of 5 *μ*m with a microtome (Leica CM1950, Germany) and subjected to H&E staining. Briefly, sections were deparaffinized with xylene, hydrated with an ethanol gradient, and washed distilled H_2_O. Then, sections were stained with hematoxylin for 2 min, washed with H_2_O for 3 min, counterstained with Eosin Y solution for 30 s, and dehydrated with serial ethanol gradients. Finally, the sections were cleaned with xylene, air dried, and mounted on a slide for observation. Oil red O staining was used to detect lipid droplets. In brief, frozen livers were cut into sections of 5 *μ*m thickness and balanced at room temperature for 10 min. Then, the sections were fixed in 4% neutral paraformaldehyde for 30 min and washed with distilled water for 30 s. After transferring and maintaining in oil red O staining solution for 20 min and washed sequentially with 60% isopropanol and distilled water, the sections were mounted on glass slides and the nuclei were stained with hematoxylin. Finally, the sections were sealed with glycerin gelatin, and pathological changes and liver lipid distribution were observed with an inverted fluorescence microscope (Olympus IX71, Japan).

### 2.9. Measurement of TNF-*α*, IL-1*β*, and IL-6

The liver tissue was thoroughly homogenized in an ice bath. After centrifugation at 4000 rpm/min for 10 min, the supernatants were collected, and CRP, IL-6, TNF-*α*, MDA, and T-SOD were determined with specific ELISA kits according to the manufacturer's protocols.

### 2.10. Western Blot

The liver tissues were lysed in ice-cold RIPA buffer (Thermo Fisher Scientific, Waltham, MA, USA). Equal amounts of protein (40 *μ*g) were then subjected to SDS-PAGE and transferred onto a polyvinylidene difluoride membrane (Millipore, USA). After blocking with TBST containing 5% nonfat milk for 1 h at room temperature, the membranes were subsequently incubated with COX-2, iNOS, TAK1, p-TAK1, AKT, p-AKT, p-IKK*β*, JNK, and p-JNK primary antibodies and HRP-conjugated secondary antibodies (1 : 1500). Specific bands were visualized by an ECL detection system, and all the data were quantified using Image J.

### 2.11. Statistical Analysis

Experimental data and pharmacokinetic parameters are given as mean ± SEM. Statistical analysis was performed using SPSS 22.0 software and *P* < 0.05 was considered statistically significant.

## 3. Results and Discussion

### 3.1. IG Improves Body Weight Loss, Hyperglycemia, and Insulin Resistance in T2DM with NAFLD Mice

**Figure 2:**
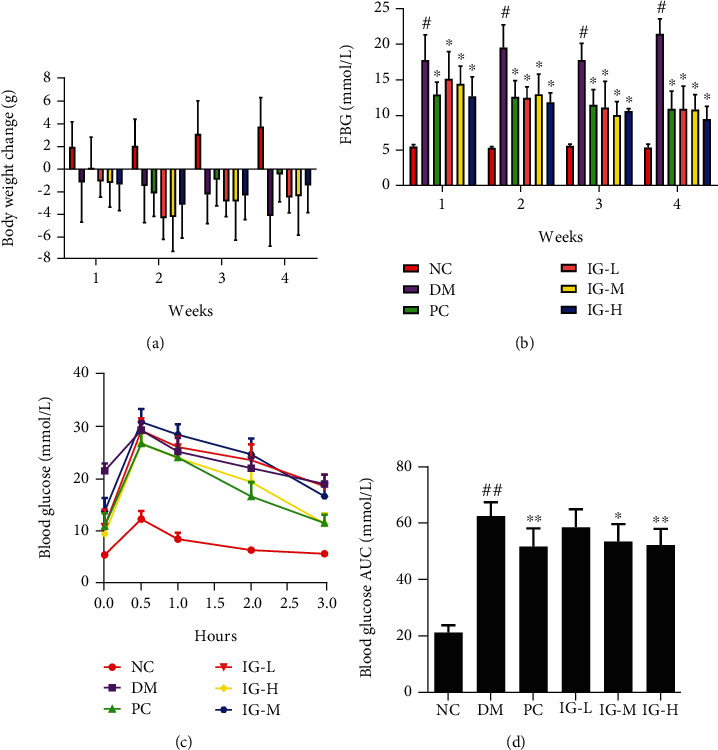
Effects of IG on body weight, FBG (fasting blood glucose), OGTT (oral glucose tolerance test), and blood glucose AUC (areas under the curve) in T2DM with NAFLD mice. (a, b) The body weight (a) and FBG (b) of the mice were measured over a four-week observation period. (c) The mice were orally administered with glucose solution at 2 g/kg BW after an overnight fasting, and blood glucose level in tail-vein sample was measured immediately prior (0 min), 30, 60, 120, and 180 min later. (d) The areas under the curve (AUC) from time zero to the last measured time were calculated, and values were expressed as mean ± SEM (*n* = 10 for each group). One-way or two-way ANOVA was carried out followed by post hoc Tukey multiple comparison test. ^#^*P* < 0.05, ^##^*P* < 0.01, ^###^*P* < 0.001 compared with the NC group; ^∗^P < 0.05, ^∗∗^*P* < 0.01, ^∗∗∗^*P* < 0.001 compared with the DM group. The data are representative of three experiments.

We reported previously that CF is a potential nutrient-rich candidate for T2DM treatment via regulating gut microbiota [24]. Among the four extracts from CF, IG apparently increased the body weight, decreased the blood glucose levels, elevated the glucose tolerance, and improved insulin sensitivity and lipid metabolism in T2DM mice induced by HFHS diet and STZ. We thus speculated that IG might play a beneficial role in T2DM with NAFLD mice. Considering body weight control is critical for diabetes, we firstly measured the body weight of the mice treated with IG weekly. As shown in Figure 2(a), the T2DM mice showed apparent weight loss as compared to stable weight gain in the normal control mice, which may be due to STZ-induced severe hyperglycaemia and nausea. However, IG treatment (especially the high dose group) gradually slowed down this effect, and animals began to regain weight. Simultaneously, IG reversed the significant increases of OGTT, FBG (*P* < 0.05), AUC (*P* < 0.05), FINS (*P* < 0.01), and HOMA-IR (*P* < 0.01) and decrease of ISI (*P* < 0.01) levels in T2DM mice, and HOMA-*β* (*P* < 0.01) was potently increased to 2.0 to 2.3-fold of the DM group after the administration of different doses of IG (Figures 2(b)–2(d), Table 1). Not surprisingly, metformin hydrochloride significantly improved the hyperglycemia of the T2DM mice, and all of them recuperated some of the weight lost as a result of STZ severity. These data collectively demonstrated that IG has a slow but stable hypoglycemic effect.

### 3.2. IG Reversed the Aberrant Lipid Metabolism in T2DM with NAFLD Mice

**Figure 3:**
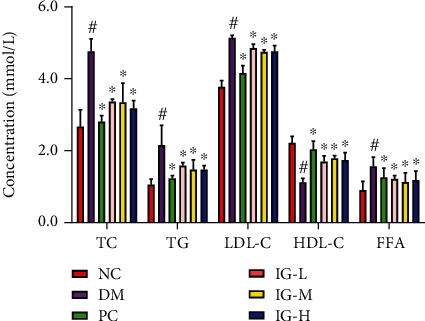
Effects of IG on TC, TG, LDL-C, HDL-C, and FFA in T2DM with NAFLD mice. Values are expressed as mean ± SEM (*n* = 10). One-way ANOVA was carried out followed by post hoc Tukey multiple comparison test. ^#^*P* < 0.01, compared with the NC group; ^∗^*P* < 0.01, compared with the DM group. Data are representative of three experiments.

Derangements in both glucose and lipid metabolic pathways exist in T2DM with NAFLD. When blood lipid levels were measured in T2DM mice, we found that IG had a similar hypolipidemic effect as metformin. Interestingly, no obvious quantitative dependence was found, and the mice treated with different IG doses obtained similar data. Compared with the NC group, the levels of TG, TC, LDL, and FFA in the DM group were significantly increased (*P* < 0.05), whereas the HDL level was significantly decreased (*P* < 0.05). After oral administration of IG, all of these indices reverted closer to the normal levels. The TC, TG, LDL-C, and FFA decreased by 29.3~33.3% (*P* < 0.05), 26.3~31.4% (*P* < 0.05), 28~39% (*P* < 0.05), and 21.8~24.4% (*P* < 0.05), respectively, and HDL level was 51.7 to 59.8% (*P* < 0.05) higher than those in the DM group (Figure 3). These data indicated that IG ameliorated the aberrant lipid metabolism in T2DM mice with NAFLD.

Consistent with our data, a few studies have uncovered the effect and mechanism of IG on aberrant lipid metabolism. For example, loganin, a major IG of CF, has been reported to exert a potent lipid-regulatory effect in the liver of type 2 diabetic mice by adjusting lipid metabolism-associated genes [25]. Swertiamarin, a bitter secoiridoid glycoside, displayed hypolipidemic activity to ameliorate NAFLD caused by hepatic lipid accumulation, inflammation, and insulin resistance via targeting potential metabolic regulators PPAR-*α* and AMPK [26]. Collectively, all these findings emphasize IG as a potentially powerful and suitable candidate for NAFLD treatment.

### 3.3. IG Treatment Improved Liver Morphology and Function in T2DM with NAFLD Mice

**Figure 4:**
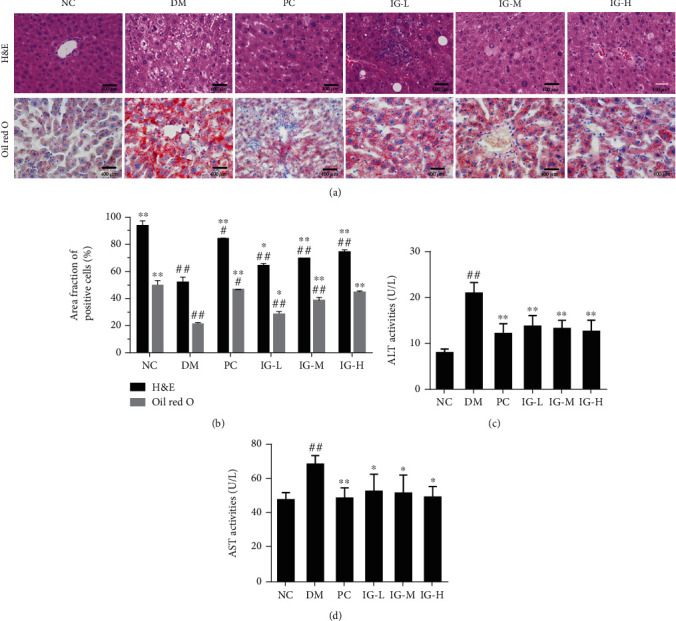
IG improves liver histological and function damage in T2DM with NAFLD mice. (a) Liver sections were prepared, and H&E and oil red staining were performed. Magnification: ×400. (b) The area fraction of positive cells was quantified by image J. (c) ALT and (d) AST activity in the liver of T2DM with NAFLD mice were detected, and values are expressed in mean ± SEM (*n* = 10 animals for each group). One-way ANOVA was carried out followed by post hoc Tukey multiple comparison test. ^#^*P* < 0.05, ^##^*P* < 0.01, compared with the NC group; ^∗^*P* < 0.05, ^∗∗^*P* < 0.01, compared with the DM group. Data are representative of three experiments.

As being a critical organ for glucose and lipid metabolism regulation, the liver with abnormal glucose uptake or lipid accumulation can trigger a wide spectrum of diseases such as insulin resistance and diabetes. Fatty liver is an important determinant in the progress of T2DM in susceptible individuals [27]. To detect the morphology changes in our study, liver sections were prepared, and H&E and Oil-red-O staining were performed. As shown in Figure 4, the liver cells were arranged uniformly, and the boundaries were clear in the NC group. In contrast, obvious hepatic lesions were observed in the liver tissues of the DM group. The lesions include an enlarged liver volume, indistinct cell boundaries, irregular lobules of liver cells, deposited fibrous tissue, and hypertrophy, vacuolization, and fatty degeneration of hepatocytes. The oil red O staining further confirmed the presence of extreme lipid accumulation. This hepatic steatosis, however, was greatly ameliorated in the IG and PC groups (*P* < 0.01), especially in the high-dose IG group as compared with the DM mice (*P* < 0.01, Figures 4(a) and 4(b)).

As to liver function, ALT and AST activities were measured, and both of them apparently increased in the DM group, which indicated that the liver function was severely damaged. Consistent with the above morphological observation, the ALT and AST activities were significantly reduced after oral administration of IG or metformin (Figures 4(c) and 4(d)).

### 3.4. IG Inhibits Inflammation in the Liver of T2DM with NAFLD Mice

**Figure 5:**
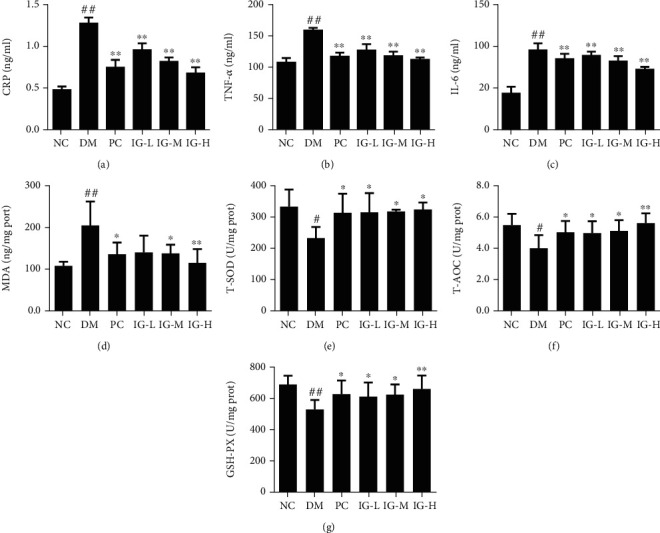
IG suppressed inflammation and oxidative stress in T2DM with NAFLD mice. (a) CRP, (b) TNF-*α*, and (c) IL-6 levels were detected by ELISA, and values are expressed in mean ± SEM (*n* = 10 animals for each group). (d) MDA, (e) T-SOD, (f) T-AOC, and (g) GSH-PX values are expressed in mean ± SEM (*n* = 10 animals for each group). One-way ANOVA was carried out followed by post hoc Tukey multiple comparison test. ^#^*P* < 0.05, ^##^*P* < 0.01, compared with the NC group; ^∗^*P* < 0.05, ^∗∗^*P* < 0.01, compared with the DM group. Data are representative of three experiments.

It is well known that T2DM is usually closely associated with low-grade chronic inflammation. In this study, inflammatory markers including CRP, TNF-*α*, and IL-6 were significantly elevated, and we found that several canonical inflammatory markers including CRP, TNF-*α*, and IL-6 were significantly elevated in the DM group as compared with the NC group. While both IG and metformin apparently reversed this phenomenon. Notably, the levels of CPR, TNF-*α*, and IL-6 suppressed by IG treatment were comparable to, if not more potent than, those inhibited by PC administration. These data suggested that IG effectively inhibited inflammation in T2DM with NAFLD mice (Figures 5(a)–5(c)).

### 3.5. IG Suppresses Oxidative Stress in T2DM with NAFLD Mice

Accumulating evidence has suggested that oxidative stress plays a critical role in the pathogenesis of diabetes [28, 29]. Therefore, T-AOC and MDA, which reflects the activities of antioxidant enzymes, were measured to indirectly evaluate the oxidative stress in mouse liver. As shown in Figures 5(d)–5(g), the MDA level was significantly increased (*P* < 0.01), whereas the levels of T-SOD (*P* < 0.05), GSH-Px (*P* < 0.01), and T-AOC (*P* < 0.05) were decreased in the T2DM with NAFLD model mice as compared to the NC group, which indicated excess hepatic oxidative stress was induced in T2DM with NAFLD mice. After oral administration of IG for 4 weeks, all these four indices tested were recovered to the normal level like the NC group. These data suggested that IG could make the T2DM with NAFLD mice regain antioxidant capacity and improve their liver injury.

### 3.6. IG Regulated NF-*κ*B and PI3K/Akt Signaling Pathways in T2DM with NAFLD Mice

**Figure 6:**
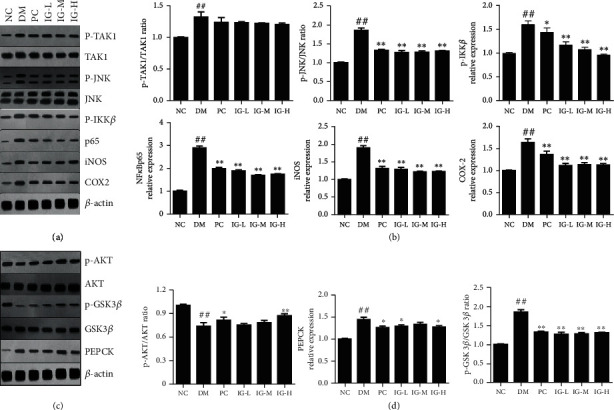
IG regulates NF-*κ*B and PI3K-AKT signal pathways. (a, b) Protein levels of p-TAK1 (phospho-T187), TAK1, p-JNK (phospho-T183 + T183 + T221), JNK, p-IKK*β* (phospho-Y199), NF*κ*Bp65, COX2, and iNOS were detected by Western blot and quantitatively analyzed. (c, d) Protein levels of p-AKT (phospho-T308), AKT, p-GSK3*β* (phospho-S9), GS3K*β*, and PEPCK were detected by Western blot and quantitatively analyzed. Values are expressed in mean ± SEM (*n* = 10). One-way ANOVA was carried out followed by post hoc Tukey multiple comparison test. ^##^*P* < 0.01 compared with the NC group; ^∗^*P* < 0.05, ^∗∗^*P* < 0.01, compared with the DM group. Data are representative of three experiments.

To uncover the mechanism that IG alleviates T2DM with NAFLD, several classical signaling pathways were detected. It is well known that TNF-*α* and IL-6 were upregulated in mice as mentioned above, which can trigger insulin resistance via activating JNK and IKK-*β*/NF-*κ*B pathways. When measured by quantitative Western blot, p-TAK1/TAK1, p-JNK/JNK, NF-*κ*Bp65, and p-IKK*β* were greatly upregulated in T2DM with NAFLD mice when compared with the NC mice. To confirm this, we detected the expression of COX-2 and iNOS, two NF-*κ*B-downstream genes extensively involved in impaired vascular function and reactive superoxide release in diabetes [30, 31]. Consistently, COX-2 (*P* < 0.01) and iNOS (*P* < 0.01) were potently induced in DM mice. IG administration significantly reduced the levels of p-JNK (*P* < 0.01), p-IKK*β* (*P* < 0.01), and NF-*κ*Bp65 (*P* < 0.01). In addition, the inhibition of p-IKK*β* expression by IG was dose-dependent. These data suggested that IG may ameliorate the inflammation of T2DM with NAFLD mice via the NF-*κ*B signaling pathway. Interestingly, no apparent effect of IG on p-TAK1/TAK1 was found in our results, which meant that the regulation of NF-*κ*B signaling pathway by IG is a TAK1 downstream event (Figures 6(a) and 6(b)).

It is reported that reduced PI3K/Akt signaling contributes to insulin resistance and T2DM [32]. In our study, p-AKT/AKT was extremely significantly decreased in the DM group as compared with the normal mice (*P* < 0.01), and both high dose IG and metformin reversed this effect. As an enzyme of the lyase family, phosphoenolpyruvate carboxykinase (PEPCK) is essential for gluconeogenesis, and the disregulation of PEPCK has been strongly suggested to be an etiologic factor in T2DM [33]. Considerable evidence suggests that the constitutively active glycogen synthase kinase 3 beta (GSK3*β*) is extensively involved in the common pathology of T2DM and could be a target for T2DM treatment [34]. As shown in Figures 6(c) and 6(d), the expression of PEPCK (*P* < 0.01) and p-GSK3*β*/GSK3*β* (*P* < 0.01) was significantly increased in DM mice, and oral administration of IG and metformin restored their levels.

## 4. Conclusions

Corni Fructus is a common health food in Asia, but also CF or its bioactive ingredients play critical pharmacological roles in some herbal prescriptions to treat various age-related diseases via its antihyperglycemic [35], memory improving [36], immune regulatory, and neuroprotective effects [35, 37]. In the present study, we demonstrated that IG extracted from CF apparently ameliorated the T2DM model with NAFLD through regulating lipid and glucose metabolism, reducing oxidative stress, inflammation, and fat accumulation in the liver. Further studies revealed that IG achieved this effect by inhibiting the NF-*κ*B signaling pathway and enhancing the activation of the PI3K/AKT signaling pathway. In short, CF or its IG extracts could be leveraged as candidates for the treatment of T2DM combined with NAFLD.

## Figures and Tables

**Table 1:** Effects of IG on the FINS, ISI, HOMA-IR, and HOME-*β* in T2DM with NAFLD mice.

	FINS	ISI	HOMA-IR	HOMA-*β*
NC	8.93 ± 1.27	−3.87 ± 0.14	2.14 ± 0.3	94 ± 13.35
DM	16.24 ± 2.03^##^	−5.85 ± 0.12^##^	15.47 ± 2.03^##^	18.12 ± 2.27^##^
PC	13.2 ± 0.51^∗∗^	−4.97 ± 0.04^∗∗^	6.37 ± 0.24^∗∗^	35.88 ± 1.37^∗∗^
IG-L	13.53 ± 0.45^∗∗^	−4.99 ± 0.03^∗∗^	6.53 ± 0.22^∗∗^	36.83 ± 1.23^∗∗^
IG-M	12.66 ± 2.56^∗∗^	−4.89 ± 0.2^∗∗^	5.98 ± 1.21^∗∗^	38.52 ± 7.19^∗∗^
IG-H	12.4 ± 1.93^∗∗^	−4.75 ± 0.16^∗∗^	5.19 ± 0.8^∗∗^	42.1 ± 6.53^∗∗^

Fasting insulin (FINS), insulin sensitivity index (ISI), insulin resistance index (HOMA-IR), and insulin secretion index (HOMA-*β*) were calculated as previously described. Values are expressed as mean ± SEM (*n* = 10). One-way ANOVA was carried out followed by post hoc Tukey multiple comparison test. Values are represented statistically when ^#^*P* < 0.05, ^##^*P* < 0.01, in comparison with the NC group; ^∗^*P* < 0.05, ^∗∗^*P* < 0.01, in comparison with the DM group. Data are representative of three experiments.

## Data Availability

The data supporting the findings of this study are available from the corresponding authors upon request.
